# Efficacy of cabergoline add-on therapy in patients with acromegaly resistance to somatostatin analogs treatment and the review of literature

**DOI:** 10.20945/2359-3997000000481

**Published:** 2022-05-25

**Authors:** Muhammed Kizilgul, Hakan Duger, Narin Imga Nasiroglu, Erkam Sencar, Sema Hepsen, Pinar Akhanli, Dilek Berker, Erman Cakal, Hayri Bostan, Bekir Ucan

**Affiliations:** 1 University of Health Sciences Diskapi Training and Research Hospital Department of Endocrinology and Metabolism Ankara Turkey University of Health Sciences, Diskapi Training and Research Hospital, Department of Endocrinology and Metabolism, Ankara, Turkey; 2 University of Health Sciences Numune Training and Research Hospital Department of Endocrinology and Metabolism Ankara Turkey University of Health Sciences, Numune Training and Research Hospital, Department of Endocrinology and Metabolism, Ankara, Turkey

**Keywords:** Acromegaly, treatment, cabergoline

## Abstract

**Objective:**

It is reported that adding cabergoline to somatostatin analog (SSA) normalizes IGF-1 levels approximately in one-third of patients with acromegaly. We investigated the effect of combination therapy and potential predictors of response in patients with acromegaly who do not respond to SSA therapy alone.

**Subjects and methods:**

Fifty acromegaly patients (M/F 23/27, mean age 50.88 ± 12.34 years) were divided into two groups as the active and control groups in this connection. Before and after treatment, we not only evaluated serum GH and IGF-1 levels and tumor size but also analyzed the factors relevant to the effect of the combined therapy.

**Results:**

Adding cabergoline to SSA treatment led to IGF-1 normalization in 42% (21/50) of patients. Mean GH levels decreased from 2.64 ± 1.79 to 1.34 ± 0.99 ng/mL (*p* < .0001) and IGF-1 levels decreased from 432.92 ± 155.61 to 292.52 ± 126.15 ng/mL (*p* < .0001). GH and IGF-1 reduction in percent (%) were significantly higher in the controlled group (63% to 40%, *p* = 0.023 and 45% to 19%, *p* = 0.0001). Moreover, tumor size decrease was significantly higher in controlled group (-3.6 cm to -1.66 cm, p = 0.005).

**Conclusions:**

According to the results of our study, the addition of cabergoline to SSA normalized IGF-1 levels in a considerable amount of acromegaly patients with a moderately elevated IGF-1 level, regardless of serum PRL levels. Besides, cabergoline treatment was also influential in patients with higher IGF-1 levels despite a lower remission rate.

## INTRODUCTION

Acromegaly is a rare and slowly progressing disease derived from overproduction of GH and, consequently, IGF1, induced by a pituitary adenoma in most of the patients. The main objectives of treating acromegaly are to normalize GH and IGF-1 levels, control tumor mass, and decrease comorbidities. However, surgery, medical therapy, and radiotherapy are provided as treatment options (1). Somatostatin analogs (SSAs) are the first-line treatment option when it is impossible to control the disease through surgery. However, at least 45% of patients are considered to be resistant to SSA treatment as they fail to achieve IGF-1 normalization (2). Cabergoline, a potent dopamine agonist, is an ergot derivative used in the treatment of hyperprolactinemia (3). Dopamine decreases GH secretion (4) and dopamine agonists inhibit GH overproduction in acromegaly patients, which have been supported by in-vitro studies conducted in GH-producing adenomas (5,6). Both GH-producing adenomas and mixed PRL-GH-producing adenomas have binding sites for dopamine (7,8). Cabergoline is recommended to patients with mild IGF-1 elevations after TSS as a first-line medical treatment or as an add-on to SSA when SSA remains ineffective alone (9). The response to combined therapy may also be explained by possible hetero-oligomerization of dopamine receptor D2R and somatostatin receptor SSTR5 to create a novel receptor with increased functional activity (10). We, here, presented a retrospective cohort study in which the efficacy of cabergoline treatment added to SSA therapy in acromegaly patients resistant to SSA therapy alone was evaluated.

## SUBJECTS AND METHODS

### Subject selection and measurements

The medical records of 324 outpatients with acromegaly in Endocrinology and Metabolism Department of Dışkapı and Numune Training and Research Hospitals were reviewed retrospectively based on the data between 2010 and 2019. Our study was made up of 50 acromegaly patients with complete medical records. Elevated IGF-1 levels despite SSA therapy (30-40 mg octreotide LAR or 120 mg lanreotide) for at least six months were the primary inclusion criteria. We know radiotherapy induces tumor reduction and hormonal control over time, hence 3 patients having received radiotherapy were excluded from this study. Before conducting our study, we obtained both ethics committee approval and written informed consent of participants.

Thirty-two patients were on octreotide LAR (long-acting repeatable octreotide) treatment and 16 patients were on Lanreotide (Somatuline Autogel). 0.5 mg per week cabergoline was started on and the weekly dose was increased gradually by 0.5-1.0 mg until IGF-1 levels examined every six weeks became normal or any side effect was observed.

GH and IGF-1 reduction were calculated according to difference between the basal levels (just before the adding of cabergoline to SSA treatment) and the levels at the last visit for patients who still use cabergoline. Guidelines recommend considering cabergoline as first-line or in addition to 1st generation SSA in those with IGF-1 < 2.5 times the upper limit of normal levels (ULNR), with the greatest benefit seen in those with an IGF-1 ULNR ≤ 1.5 (9,11).

Before and after treatment, we not only evaluated serum GH and IGF-1 levels and tumor volume but also analyzed the factors relevant to the impact of the combination treatment. An unequivocally increased serum IGF-1 concentration with typical clinical manifestations, inadequate suppression of serum GH after 75 g glucose loading test, and the presence of a pituitary tumor were used to diagnose acromegaly. Remission criteria were described as normal age and gender-adjusted normal ranges circulating IGF-1 level and a postoperative random GH level of < 1 ng/mL or GH level of < 0.4 ng/mL after a glucose load. The patients who achieved age and gender-adjusted IGF-1 levels normalization after cabergoline treatment and who were with persistent increased IGF-1 levels were considered to be with controlled and active diseases, respectively. In cases with discrepant GH and IGF-1 levels, only IGF-1 values were used for the decision of remission as recommended (2,12).

The GH and IGF-1 concentrations were measured by using chemiluminescence on an IMMULITE 2000 Xpi (Siemens Healthcare Diagnostics Inc.). Serum IGF-1 levels were compared with the age-sex adjusted normal reference values obtained from manufacturer’s instructions for use. IGF-1 levels were also expressed as “x ULNR (upper lower normal range)” due to differences in normality range across different ages.

A two-site chemiluminescent immunometric assay was used to measure serum prolactin (PRL) (Immulite 2000). The normal range for prolactin was as follows: 2.0-17.0 ng/mL in males and 4.6-21.4 ng/mL in females. The inter-assay coefficient of variation was less than 5%. The presence of prolactin levels above the normal range corresponds to hyperprolactinemia. The immunohistochemical (IHC) features of the tumors were available in 45 patients previously submitted to surgery. Hormonal positivity was established if at least 10% of the cells showed immunopositivity.

The largest tumor diameter on MRI was accepted as tumor size. Tumor volume was calculated using the ellipsoid formula ( volume=height×width×depth×0.52), as previously described (13). An echocardiography was performed at baseline and annually (if abnormal) in accordance with the guidelines (14).

### Statistical analysis

All statistical analyses were made by using JMP 14.0.1 software (SAS Institute, Cary, NC, USA). Mean ± standard deviation was used for the expression of quantitative data, whereas counts and proportions were used for the expression of categorical data. The Kolmogorov-Smirnov or Shapiro-Wilk W test was used to test the normality of distribution. Categorical variables were examined by means of the Chi-square or Fisher’s exact test. Normally distributed continuous variables were analyzed through Student’s t-test whereas non-normally distributed variables were analyzed through the Mann-Whitney U test. Matched paired t-test was used to detect a pre-post difference with cabergoline treatment. Pearson’s and Spearman’s correlation were preferred to assess correlations. A *p*-value less than 0.05 was accepted as statistically significant.

## RESULTS

### Characteristics of the patients

Treatments before the addition of cabergoline included trans-sphenoidal surgery (TSS) and SSA in 49 patients (98%) and primary therapy with SSA in 1 patient (2%). The effect of cabergoline as an add-on therapy was evaluated in a cohort of 50 patients (M/F 23/27, mean age 50.88 ± 12.34 years) with acromegaly. Adding cabergoline to SSA treatment led to IGF-1 normalization in 42% (21/50) of patients. Median (IR) for GH and IGF-1 levels before the beginning of combined therapy were 1.95 (2.64) and 396 (105.75), respectively. Additionally, hyperprolactinemia was present in 11 of 50 patients, and mean prolactin levels were 19.26 ± 19.69.

Forty-seven patients who were previously performed TSS had the immunohistochemical (IHC) features of the tumors. The whole adenoma stained positive for GH and 15 of them (30%) were positive for PRL (Tables 1 and 2). Macroadenoma remnants were detected through pituitary MRI in 24 of 50 patients undergone TSS. All patients were on SSA therapy before the addition of cabergoline. Visual field defects were not observed in any patients.

**Table 1:** Characteristics of patients on adjuvant treatment with somatostatin analog plus cabergoline

	Active (n = 29)		Controlled (n = 21)		*p*
	Median or n	IR or %	Median or n	IR or %	
Females, n (%)	17	59	11	52	0.441
GH before treatment (mIU/L)	2.37	2.82	1.90	1.94	0.331
GH reduction (%)	40.00	52.18	63.16	45.01	**0.023**
IGF-1 before treatment (nmol/L)	427.00	262.50	360.00	93.50	**0.001**
xULNR for IGF-1	1.92	0.66	1.39	0.35	**<.0001**
IGF-1 reduction in (%)	19.71	27.72	44.87	24.96	**<.0001**
Median dose (mg/week)	1.50	1.00	1.25	1.00	0.992
Microadenom, n (%)#	12	41	11	52	0.441
Duration of treatment in months	48	51	48	66	0.992
Hyperprolactinemia, n (%)	6	22	5	25	0.824
Basal Prolactin, (ng/mL)	12.00	13.45	14.24	22.80	0.232
Prolactin reduction (%)	95.71	9.01	95.75	9.76	0.665
Positive IHC staining for PRL##	6	22	9	45	0.098

# Adenoma size before cabergoline treatment. 1 patient with empty sella and 1 patient with no lesion on MRI were categorized into microadenoma group.

## Immunohistochemistry findings were available for 47 patients.

**Table 2:** Comparison of hormone levels during treatment with the combination of SSA/cabergoline

	Before Cabergoline Treatment	After Cabergoline Treatment	Change	*p*
Mean GH levels (ng/mL)	2.64 ± 1.79	1.34 ± 0.99	-1.29	**<.0001**
Mean IGF-1 levels (ng/mL)	432.92 ± 155.61	292.52 ± 126.15	-147.4	**<.0001**
Mean PRL levels (ng/mL)	19.26 ± 19.69	1.47 ± 3.07	-17.79	**<.0001**

### Efﬁcacy of adding cabergoline

A significant decrease was observed in mean IGF-1, PRL, and GH levels following cabergoline add-on therapy. Mean GH levels decreased from 2.64 ± 1.79 to 1.34 ± 0.99 ng/mL (p < .0001) and IGF-1 levels decreased from 432.92 ± 155.61 to 292.52 ± 126.15 ng/mL (p < .0001). Mean xULNR for IGF-1 was significantly decreased after cabergoline add-on therapy (1.74 ± 0.61 to 1.18 ± 0.52, p < .0001). The median xULNR for IGF-1 level was found to be lower in the controlled group compared to the active group. (1.39 (0.35) to 1.92 (0.66) p < .0001). The mean duration of cabergoline use was 55.5 ± 34.09 months. Cabergoline dose ranged from 0.5 to 10 mg/week (median (IR) 1.5 ± 1 mg/week). Twenty-one (42%) patients achieved normal IGF-1 levels after cabergoline add-on therapy and the mean decrease in IGF-1 levels in the rest of the patients was 139 ± 31.06 (p < 0.0001). Furthermore, serum levels of GH lower than 1 ng/mL after cabergoline treatment were observed in 26 patients (52%). The disease remission (normal IGF-1 levels and safe GH levels) with the combined therapy was obtained in 16 of 50 patients (32%).

The final mean IGF-1 levels were 197.52 ± 60.69 (min-max, 100-281) in the controlled group but 361.31 ± 116.441 (min-max, 221-633) in the active group. GH and IGF-1 reduction in percent (%) were significantly higher in the controlled group (63% to 40%, *p* = 0.023 and 45% to 19%, *p* = 0.0001) (Table 1). Mean percentage reduction (%) in IGF-1 and GH level was 32 and 34, respectively, after the combined therapy.

Prolactin levels also decreased signiﬁcantly during cabergoline treatment (19.26 ± 19.69 to 1.47 ± 3.07 ng/mL, *p* < 0.0001). Hyperprolactinemia was present in 11 of 50 patients at the beginning; however, normal serum PRL levels were achieved in all patients after cabergoline treatment. Positive expression of the PRL in the tumor was observed in 7 of these 10 patients, which may suggest co-secretion of PRL as the reason behind hyperprolactinemia. Stalk compression could be the reason for hyperprolactinemia in the rest of the patients.

Mean cabergoline dose was found to be lower in patients with the controlled disease, although it did not reach a statistically significant point (1.55 ± 0.77 to 1.83 ± 1.71, p:0.434). Adding cabergoline to SSA therapy was tolerated well and no patients had to discontinue the therapy. Moreover, cardiac valve regurgitation assessed through an echocardiogram during treatment was not observed in any patients.

Tumor size decreased with combined treatment in all patients (11.91 ± 7.34 to 9.43 ± 7.24, *p* < .0001). The decrease in tumor size with combined treatment was higher in controlled group when compared to active group (-3.6 cm to -1.66 cm, *p* = 0.005). Tumor volume decreased with combined treatment (710.92 ± 1020.38 to 541.57 ± 864.75, *p* = 0.002).

### Correlation of change in IGF-1 during combined therapy with baseline parameters

Compared to both groups, baseline prolactin level and the rate of hyperprolactinemia did not significantly differ (17.73 ± 14.14 to 20.34 ± 23.01, *p* = 0.637, and 21% to 22%, *p* = 0.924). Although the remission rate in patients with positive expression of the PRL in the tumor was higher, it did not reach a statistically significant point [6 (22%) vs. 9 (45%); *p* = 0.098] (Table 1).

We also revealed that 16 of 21 controlled patients (76%) had GH levels up to 2.5 ng/mL. 13/29 (45%) of non-responsive subjects presented GH levels > 2.5 ng/mL by contrast. Besides, IGF-1 levels of 16 (50%) of 32 patients with GH ≤ 2.5 ng/mL returned to normal during cabergoline add-on therapy. Finally, 5 of 18 patients with GH>2.5 ng/mL and 16 of 32 patients with GH ≤ 2.5 ng/mL achieved remission with cabergoline add-on treatment (*p* = 0.121). Remission rate was higher in patients with IGF-1 ≤ Upper limit of normal range (ULNR)-IGF-1 220% when compared to patients with IGF-1 > ULNR-IGF-1 220% (9 (82%) to 12 (31%), *p* = 0.002). The lowest rate of IGF-1 normalization (4/17, 23%) was observed in the subgroup of patients with % ULNR-IGF-1 > 220% and random GH levels were > 2.5 ng/mL. The decrease in IGF-1 levels in patients with IGF-1 > ULNR-IGF-1 220% and with IGF-1 ≤ ULNR-IGF-1 220% were similar (*p* = 0.399).

The decrease in serum IGF-1 levels during combined therapy was not correlated with basal GH, IGF-1, and PRL levels, the tumor size before cabergoline add-on therapy (Table 3). Mean decrease in IGF-1 levels was found to be higher in the remission group (-159 to -139.6, *p* < 0.0001).

**Table 3:** Correlation of change in IGF-1 during combined therapy with baseline parameters

	r	*p*
Baseline GH	-0.229	0.109
Baseline IGF-1	0.077	0.594
Baseline Prolactin	0.103	0.488
Tumor size before combined therapy	0.071	0.631

## DISCUSSION

As a consequence of our study, we revealed that IGF-1 levels decreased significantly following cabergoline add-on therapy in acromegaly patients resistant to SSA therapy alone. IGF-1 levels reduced by 32% compared with baseline and IGF-1 normalization achieved in 42% (21/50) of patients. Moreover, a substantial reduction in tumor size was observed as a result of combined therapy.

The disease control in acromegaly is determined with an age-sex normalized serum IGF-1 value or a random GH < 1.0 µg/L (1). IGF-1 levels are correlated with comorbidities better than suppressed GH levels after glucose load (15); however, it is indicated that normalized IGF-1 and random GH level lower than 1.0 < g/L are correlated with mortality risk reductions (16,17). Surgery is preferred as the usual first-line treatment in acromegaly. The success rate of surgery (microscopic or endoscopic trans-sphenoidal microsurgery) is around 85% for microadenomas and 40%-50% for macroadenomas (18,19). Second or third surgery can be considered in a patient with disease recurrence after the first surgery (1). When the normal GH/IGF-1 levels are not achieved in acromegaly patients after surgery, either SSA or pegvisomant is recommended as an adjuvant treatment/therapy (1). The accuracy of glucose-suppressed GH values during treatment with SSAs was not clear (20); hence we used age-sex normalized serum IGF-1 value for disease control in our study. IGF-1 levels became normal in 30%-55% of patients using SSAs after the surgery fails (2).

Cabergoline has been preferred in order to treat not only hyperprolactinemia but also Parkinson’s disease for almost thirty years as it is one of ergot-derivative dopamine agonists (3). GH-secreting adenomas may contain dopamine receptors and cabergoline has also been proven effective in acromegaly (8). Cabergoline binds to pituitary dopamine type 2 (D2) receptors and suppresses GH secretion while the precise mechanism of its action remains unclear. Dopamine agonist treatment of cultured somatotropic adenoma decreased GH secretion by 20%-25% or more (21,22) while a resistance was observed in tumors without dopamine receptor expression (23). Biochemical control with cabergoline alone was achieved in approximately one-third of acromegaly patients according to a meta-analysis. The effect of cabergoline was not related to the duration of cabergoline treatment and the dose of the medicine or baseline prolactin levels but baseline IGF-1 levels (3). In our study, the lower baseline IGF-1 levels ensured better remission rate. However, baseline prolactin levels, tumor size and the rate of hyperprolactinemia did not affect the remission rate.

Patients with mildly increased levels of GH and IGF-1, with or without hyperprolactinemia, are more likely to have benefited from cabergoline treatment. Although cabergoline is effective at the beginning of the treatment, its effect may decrease in time. In a study, only 21% of subjects had disease control with an 18-month treatment of cabergoline (24). According to a large prospective study, IGF-1 normalization was achieved in 39% (25/64) of patients treated with cabergoline monotherapy (at doses ranging 1.0-3.5 mg weekly) for 3-40 months. Additionally, patients with GH/PRL-secreting adenomas had a higher rate of IGF-1 normalization (50%), and tumor shrinkage was detected in 62% (13/21) of patients (11). However, the lack of age-adjusted normal IGF-1 values was a limitation of this study. Vilar and cols. demonstrated that cabergoline (1.0-3.0 mg/week) add-on therapy in acromegaly patients resistant to SSA therapy alone could achieve IGF-1 normalization in 40.4% of cases (25). A recent meta-analysis has been revealed that cabergoline single-agent therapy normalizes IGF-1 levels in more than one-third of acromegaly patients. When SSA is ineffective, the addition of cabergoline normalizes the IGF-1 level in 40%-50% of patients. Moreover, patients with moderate elevations of IGF-1 levels (1.5 × ULN) were more likely to benefit from cabergoline add-on therapy (26). According to UK Acromegaly Register from 31 centers at which 41.4% of the whole patient used cabergoline, both GH and IGF-1 normalization was achieved in 26% of patients on cabergoline monotherapy and in 20% of patients with cabergoline add-on therapy (27). The results of Bulgarian Acromegaly Database have revealed that cabergoline monotherapy normalizes IGF-1 levels in more than one-third of acromegaly patients, however, better remission rate was observed in previously irradiated patients (28). Fifty-eight percent remission rate following cabergoline add-on therapy and better results have been reported in a recent study conducted in Turkey, particularly in patients with acromegaly having small adenomas and not having fibrous bodies (29). Studies, which investigate the effect of cabergoline add-on therapy, provided variable results, and these studies were mostly small-sized. Furthermore, none of these studies was randomized placebo-controlled (3). These findings may lead to reach a general idea that cabergoline had a mild effect on patients or it was only effective in patients with modest elevation of IGF-1 level or tumors that secrete both GH and prolactin (30). Patients with low IGF-1 levels demonstrated a better response (25). IGF-1 normalization was associated with basal IGF-1 < 2.2 ULNL and/or basal GH < 5 ng/mL in patients with cabergoline add-on therapy (25,31). Moreover, the rate of IGF-1 normalization in acromegalic patients with %ULNR IGF-1 ≤ 220% ranged between 41%-86% according to the previous studies (25). In our study, the remission rate was lower (23%) in patients with higher baseline IGF-1 and/or GH levels > 2.5 ng/mL. Additionally, the rate of IGF-1 normalization in patients with %ULNR IGF-1 ≤ 220% was 82% in our study.

Although there was no correlation between the decrease in IGF-1 levels during the combined therapy and pre-treatment levels of IGF-1, mean decrease in IGF-1 levels were higher in the remission group. IGF-1 level pre-treatment (<220% ULRN) were predictive for a better response and the remission group had lower pre-treatment IGF-1 levels. The higher pre-treatment IGF-1 levels in the active group might be due to more aggressive disease and less possibility of response to cabergoline add-on therapy. The remission rate in patients with positive expression of the PRL in the tumor was higher; however, it did not reach a statistically significant point. Although it is not statistically significant, more patients with positive expression of the PRL in the tumor in the remission group may also contribute to better results in those patients.

Various studies evaluating the effect of cabergoline treatment on tumor volume found tumor shrinkage in some patients (11,32,33). Sandret and cols. found greater tumor shrinkage in patients with higher baseline prolactin and IGF-1 levels (3). Suda and cols. reported that combined therapy was effective for the decrease in tumor volume (34). However, it is difficult to differentiate whether ongoing treatment with SSA or cabergoline add-on therapy was influential in the reduction of tumor volume at least in some cases. We found a decrease in tumor size with combined therapy. Although the decrease in IGF-1 levels was not correlated with a reduction in PRL level and tumor size, in the controlled group, the tumor size was found to be larger than the other group. It was shown that an improved response to cabergoline alone was correlated with the presence of hyperprolactinemia in a large number of acromegaly patients (11,35-38). IGF-1 normalization rate with SSA and cabergoline combined therapy was not influenced by PRL status according to the previous studies (31,35-37). Vilar and cols. notified that response to the combined therapy was not dependent on the presence of hyperprolactinemia or prolactin staining in tumor cells by immunohistochemistry (25). In our study, immunohistochemical prolactin expression on the tumor was present in 14 out of 48 patients. Neither the presence of hyperprolactinemia nor the positive immunohistochemical staining for prolactin was related to the reduction in IGF-1 or the rate of disease control, supposing that presence of PRL expression might not predict the response. The range for cabergoline dose was between 1 and 3.5 mg/week (mean dose, 2.2 mg/week) according to the previous studies (Table 4) (34). The mean cabergoline dose was 1.7 mg/week in our study.

**Table 4:** Characteristics of the published studies evaluating the effect of combined therapy in patients with acromegaly

	N	RT	IHC positive PRL	Elevated PRL, n/N	SSA type OCT, SR or LA	SSA mean duration (month)	CAB dose (mg/week)	CAB mean duration (month)	GH normalization, % (n/N)	IGF-1 normalization, % (n/N)
Marzullo *et al*.	10	0/10	5/10	2/10	SR 10/10	6	1.5-3 mg	3	40% (4/10)	50% (5/10)
Cozzi *et al*.	19	2/19	4/8	2/19	ATG:6, OCT:13	9-12	1-3.5 mg	3-18	21% (4/19)	42% (8/19)
Gatta *et al*.	9	5/9	3/6	0/9	ATG:1, OCT:8	27	1.8 mg	8.44	44% (4/9)	44% (4/9)
Jallad *et al*.	34	14/34	11/21	13/34	OCT 34/34	24	1.5-3.5 mg	6	71% (24/34)	56% (19/34)
Mattar *et al*.	19	4/19	4/9	0/19	OCT 19/19	24	1-3.5 mg	4-12	NA	37% (7/19)
Vilar *et al*.	52	6/52	7/15	17/52	OCT 52/52	12	1-3 mg	6-12	46% (24/52)	40% (21/52)
Suda *et al*.	10	2/10	3/4	5/10	OCT 10/10	36	1-2 mg	6	20% (2/10)	30% (3/10)
Vandeva *et al*.	20	-/20	NA	NA	OCT 20/20	NA	2 mg	17.1	NA	35% (7/20)
Kasuki *et al*. #(40)	62	13/62	NA	23/50	NA	NA	2.5 mg	34	42% (26/62)	52% (32/62)
Kadioglu *et al*.	129	43/129	26/97	NA	OCT:95 LA:34	NA	2.87 mg	58.84	NA	58% (75/129)
Present Study	50	0/48	15/50	11/50	OCT:31 LA:19	18	1.7 mg	55	52% (26/50)	42% (21/50)

IHC: immunohistochemistry; SSA: somatostatin analog; OCT: octreotide LAR; SR: lanreotide slow-release; LA: lanreotide autogel; CAB: cabergoline; NA: not applicable.

* 49 of 56 patients had GH level of > 1 ng/mL.

# 43 (69%) patients underwent surgery prior to cabergoline add-on treatment. Median SSA dose was 20 mg (range 20-30).

Cabergoline is recommended to the patients with mild IGF-1 elevations after TSS as a first-line medical treatment or as an add-on to SSA when it remains ineffective alone (9). Increasing SSA dose and adding cabergoline or pegvisomant to SSA treatment are several options for medical therapy in the management of acromegaly patients not responding to SSA treatment alone. Although adding pegvisomant is more effective than cabergoline, pegvisomant is an expensive treatment choice and may cause transient liver enzyme elevation in up to one-third of patients (39). Our findings revealed that the combined therapy is influential in SSA resistant acromegaly cases, particularly in those patients with slightly elevated IGF-1 levels.

A considerable number of patients with long-term follow-up data and concurrent assessment of radiology, pathology, and biochemistry parameters are strengths of our study. Nevertheless, our study had some limitations such as its retrospective design. Sustained control of acromegaly after long-term combined treatment could eventually be due to remission of the disease, which cannot be excluded.

In conclusion, medical treatment is generally required when surgery fails to normalize GH/IGF-1 levels in patients with acromegaly. Cabergoline is an inexpensive, well-tolerated medicine and normalizes IGF-1 levels, particularly used as an add-on therapy to SSA, in a considerable proportion of patients with a moderately elevated IGF-1, which suggests that the combined therapy deserves a more relevant role in the management of acromegaly. Cabergoline is also beneficial in those patients with higher IGF-1 levels despite lower remission rate, and the beneficial effects of cabergoline may occur even when pre-treatment prolactin levels are within the normal range.
